# Supplementary material for: Analysis of sloppiness in model simulations: Unveiling parameter uncertainty when mathematical models are fitted to data
Source: Sci Adv. 2022 Sep 21;8(38):eabm5952. doi: 10.1126/sciadv.abm5952 (PMC9491719; doi:10.1126/sciadv.abm5952)
Supplement: Supplementary file 1 — Figures’ Supplementary Legends Figs. S1 to S21 Tables S1 to S4 References [file sciadv.abm5952_sm.pdf]

Science Advances
AAAS

Supplementary Materials for

Analysis of sloppiness in model simulations: Unveiling parameter uncertainty when mathematical models are fitted to data

Gloria M. Monsalve-Bravo *et al.*

Corresponding author: Gloria M. Monsalve-Bravo, g.monsalvebravo@uq.edu.au

Sci. Adv. **8**, eabm5952 (2022)
DOI: 10.1126/sciadv.abm5952

This PDF file includes:

Figures' Supplementary Legends
Figs. S1 to S21
Tables S1 to S4
References

Figures' Supplementary Legends

Additional legend information for Figs. 2, 4, 5, 6B and Figs. S1A to S3A, S5, S7, S8, S10, S13, S14, S15A to S17A, S18 and S20

- **Shaded regions (, , , , , , , , ,):** Prior and posterior distributions for the model parameters. Probability distributions are obtained via kernel density estimation (*86*) based on the initial sample of the prior and the sample of the posterior obtained from our Sequential Monte Carlo (SMC) sampling algorithm assuming a reflecting boundary correction to account for prior bounds (*87*). The y-axes correspond to relative (rather than absolute) probabilities, with all density functions rescaled between $0 - 1$.
- **Solid vertical lines (——):** Prior distribution mean for the model parameters, estimated from from the prior distribution sample.
- **Dash-dotted vertical lines (–·–·–, –·–·–, –·–·–, –·–·–, –·–·–, –·–·–, –·–·–, –·–·–, –·–·–):** Posterior distribution mean for the model parameters, estimated from the posterior distribution sample.
- **Open circle symbols (○):** Prior distribution samples.
- **Closed circle symbols (●●●):** Posterior distribution samples, in which the color change across posterior samples represents changes in the values of the rescaled log-likelihood function with ● $= 0$ and ● $= 1$.
- **Dashed vertical lines (– – –) and plus sign symbols (+):** Set of reference (true) parameter values.
- **Dotted vertical lines (·········) and cross symbols (×):** Set of best-fit parameter values obtained via maximum likelihood estimation (MLE).

Additional legend information for Figs. 1, 3B, 6A, 7 and Figs. S1B to S3B, S9, S12, S15B to S17B, S19 and S21

- **Open triangle (△) and square (□) symbols:** Synthetic data
- **Dark (, , , , , , , ,) and light (, , , , , , , ,) shaded areas:** 68% and 95% central credible intervals for the model ensemble prediction, obtained from posterior simulation considering all plausible parameter values estimated via Bayesian inference implemented using our SMC sampling algorithm.
- **Dashed lines (– – –, – – –, – – –, – – –, – – –):** Model predictions based on the set of reference (true) parameter values.
- **Dotted lines (·········):** Model predictions based on the set of best-fit parameter values obtained maximum likelihood estimation (MLE).
- **Dashed vertical lines (— — —):** Start time of model forecasts (only applicable for the ecological network model).

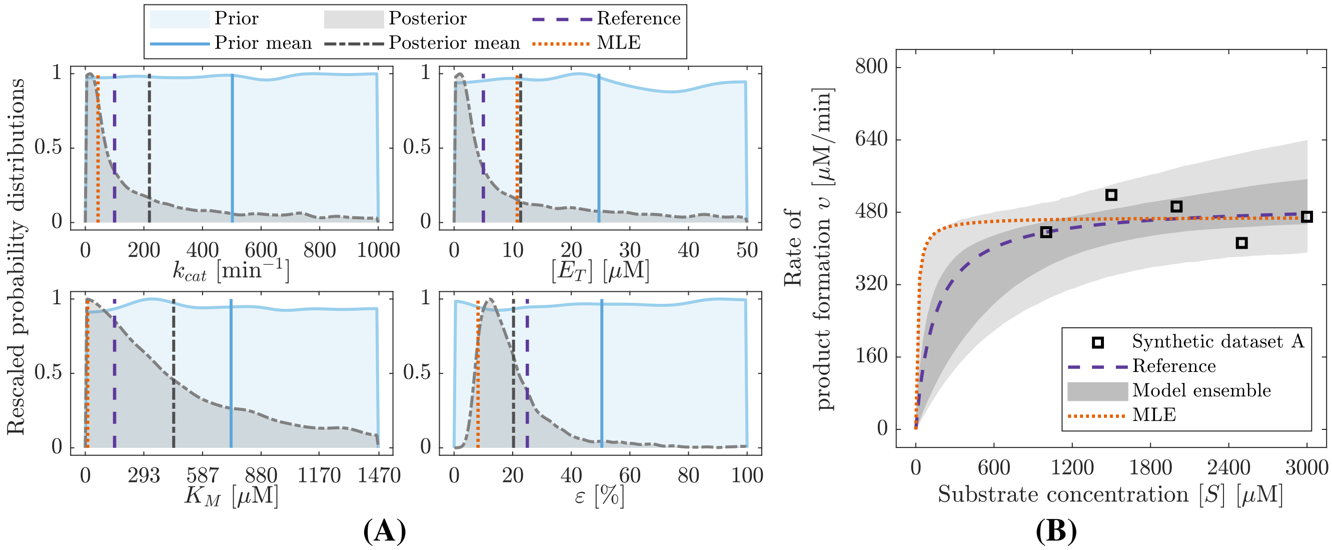

Fig. S1. Estimated parameter values together with fit of the Michaelis–Menten model to the noisy synthetic dataset A (i.e., high substrate concentration with $[S] \gg K_M$) considering uniform prior distributions for all parameters. **(A)** Prior and posterior distributions for the parameters together with reference parameter values and best-fit parameter values. **(B)** Dataset A together with noiseless model prediction using reference parameter values, model predictions using two sets of best-fit parameter values, and model ensemble predictions using all plausible parameter values obtained via Bayesian inference. While the model ensemble fits synthetic dataset A, the data is uninformative for parameter K_M. (See also Figures' Supplementary Legends.)

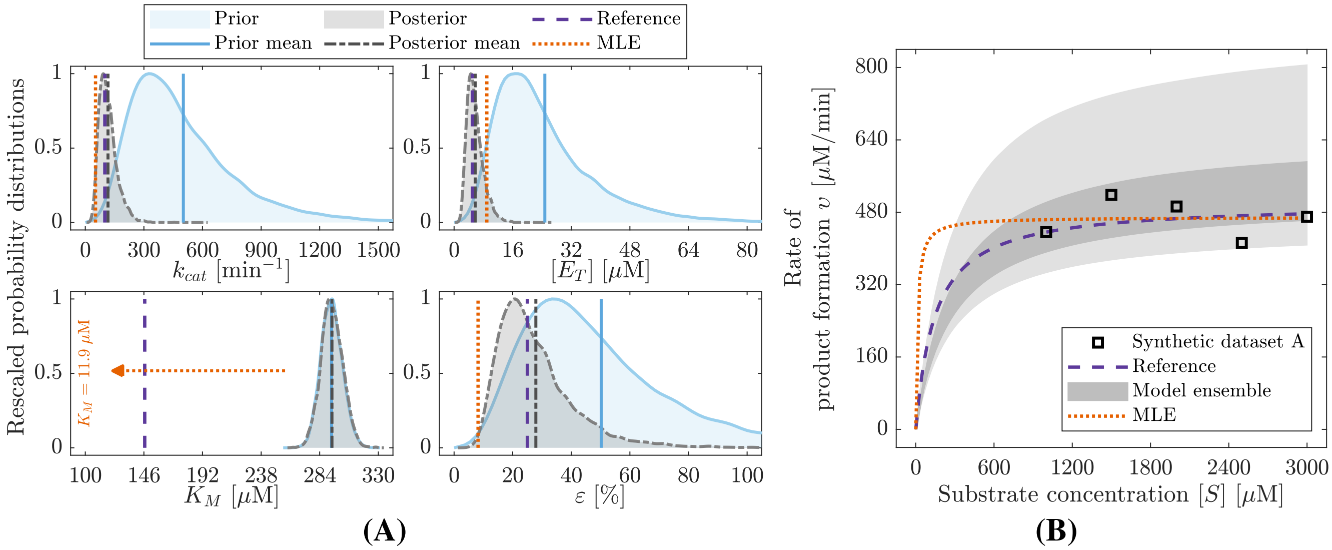

Fig. S2. Estimated parameter values together with fit of the Michaelis–Menten model to the noisy synthetic dataset A (i.e., high substrate concentration with $[S] \gg K_M$) considering a multivariate log-normal prior distribution for all parameters with that of parameter K_M badly specified with $p(K_M = 146.7\ \mu\text{M}) \approx 0$. **(A)** Prior and posterior distributions for the parameters together with reference parameter values and best-fit parameter values. **(B)** Dataset A together with noiseless model prediction using reference parameter values, model predictions using two sets of best-fit parameter values, and model ensemble predictions using all plausible parameter values obtained via Bayesian inference. While the model ensemble fits synthetic dataset A, posterior distribution and best-fit values for K_M lie far away from the reference value. (See also Figures' Supplementary Legends.)

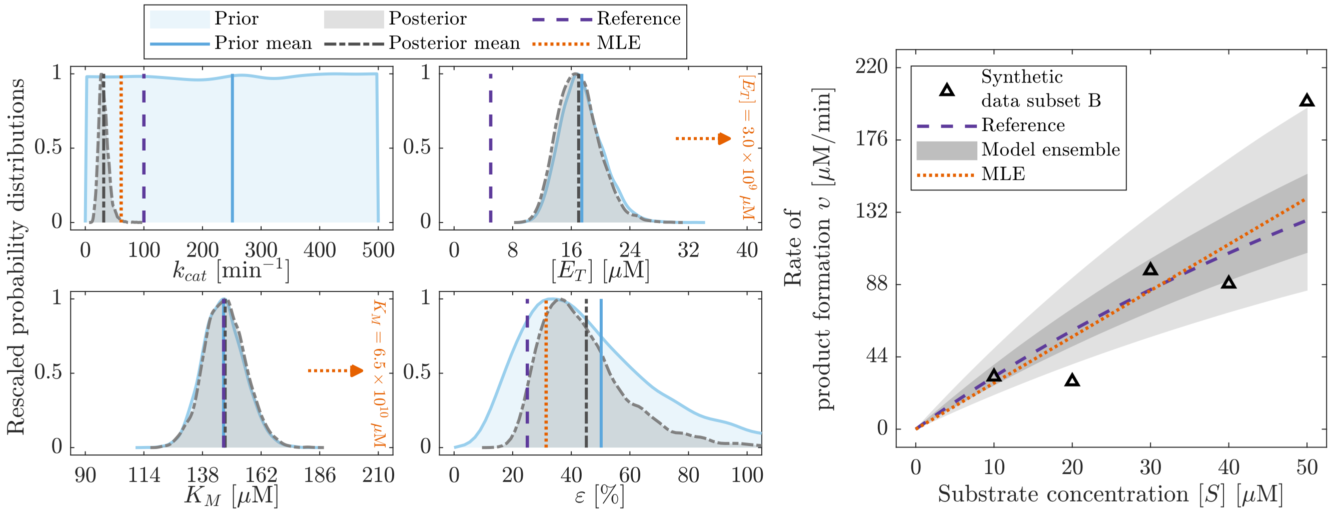

(A) **(B)**

Fig. S3. Estimated parameter values together with fit of the Michaelis–Menten model to the noisy synthetic dataset B (i.e., at low substrate concentration with $[S] \ll K_M$) considering a uniform prior for k_{cat}, a badly specified log-normal prior for $[E_T]$ with $p([E_T] = 5\ \mu\text{M}) \approx 0$, a well-specified log-normal prior for K_M with $p(K_M = 146.7\ \mu\text{M}) \approx 1$ and a log-normal prior for σ. **(A)** Prior and posterior distributions for the parameters together with reference parameter values and best-fit parameter values. **(B)** Dataset B together with noiseless model prediction using reference parameter values, model predictions using two sets of best-fit parameter values, and model ensemble predictions using all plausible parameter values obtained via Bayesian inference. While the model ensemble fits synthetic dataset B, best-fit values for parameters $[E_T]$ and $[K_M]$ lie far away from their reference values. (See also Figures' Supplementary Legends.)

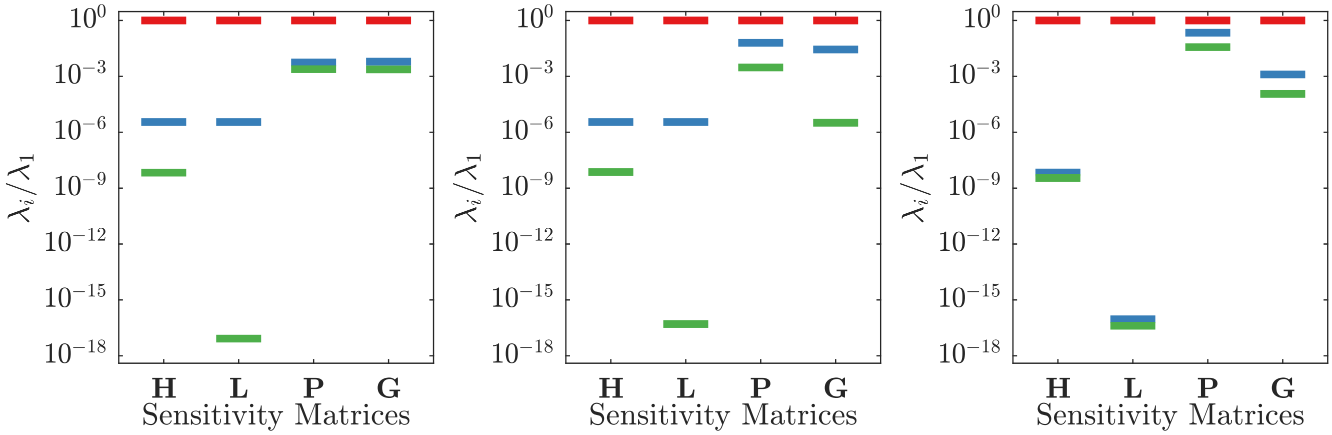

(A) **(B)** **(C)**

Fig. S4. Sensitivity matrix eigenvalue spectra (based on SVD) for the Michaelis–Menten model fit to noisy synthetic data, considering **(A)** uniform prior distributions for all parameters (Scenario 1, Fig. S1), **(B)** multivariate log-normal prior distribution for all parameters with that of parameter K_M badly specified (Scenario 2, Fig. S2), and **(C)** a uniform prior for k_{cat}, a badly specified log-normal prior for $[E_T]$, a well-specified log-normal prior for K_M (Scenario 3, Fig. S3). Largest eigenvalue λ_1 is used to rescale eigenvalues between $0 - 1$.

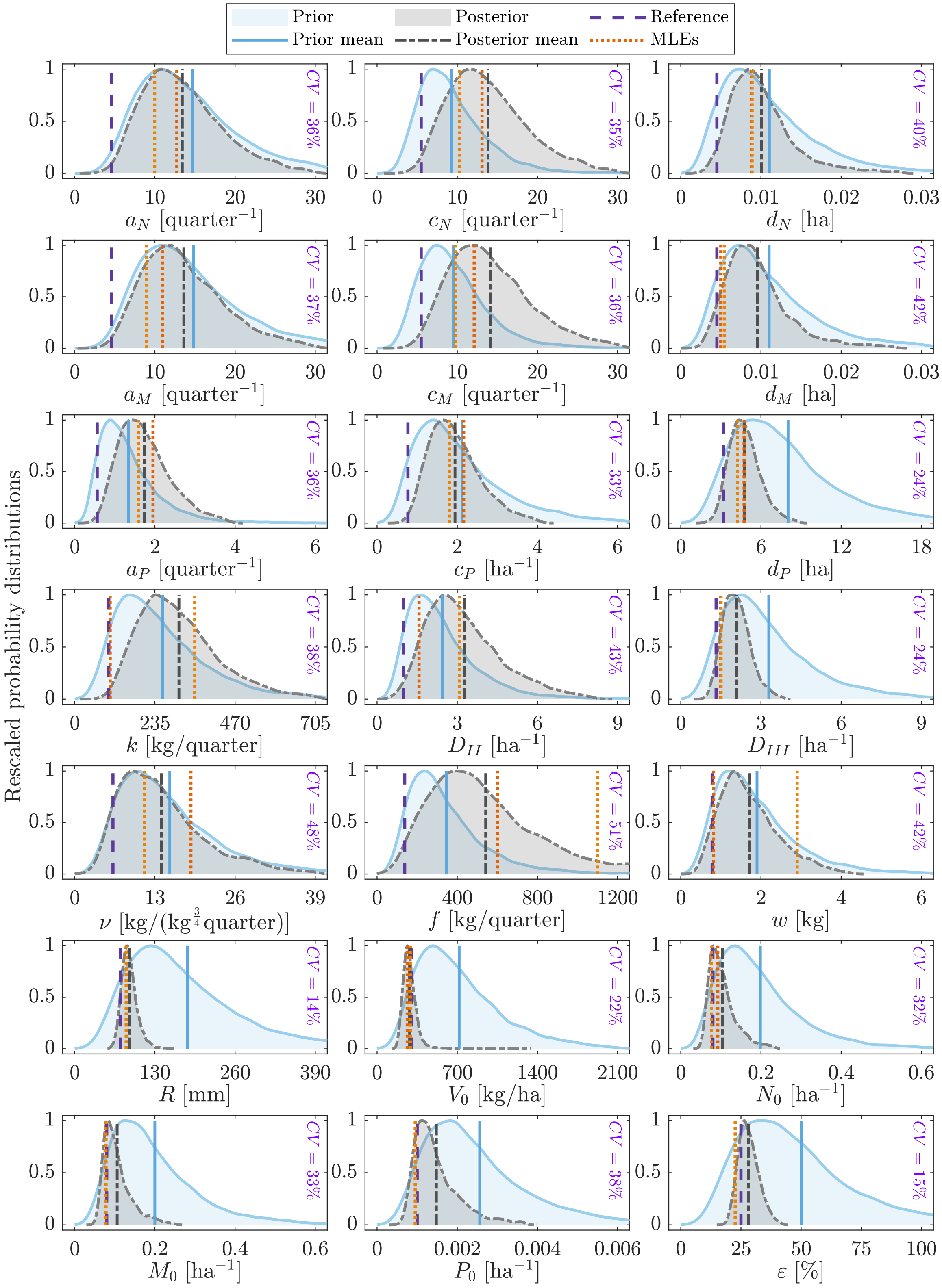

Fig. S5. Prior and posterior distributions for the parameters of the ecosystem network model (Table S1) together with the sets of reference parameter values and best-fit parameter values (MLEs) considering a vague multivariate log-normal prior for all parameters. Percentage coefficient of variation for the posterior distribution sample of each parameter (CV) is reported within each panel (compare to Fig. S8). Most of the model parameters are poorly constrained by the data. (See also Figures' Supplementary Legends.)

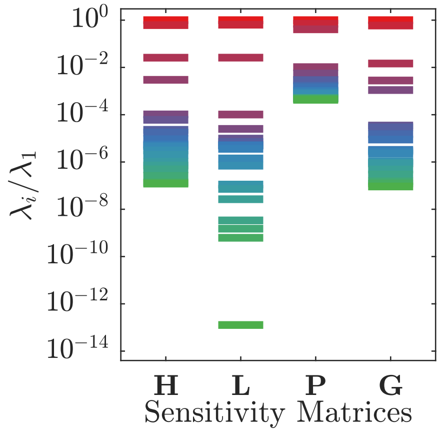

Fig. S6. Sensitivity matrix eigenvalue spectra (based on SVD) for the ecosystem network model in Table S1 fit to noisy synthetic data, considering a multivariate log-normal prior distribution for all parameters (Fig. S5). Largest eigenvalue λ_1 is used to rescale eigenvalues between $0-1$.

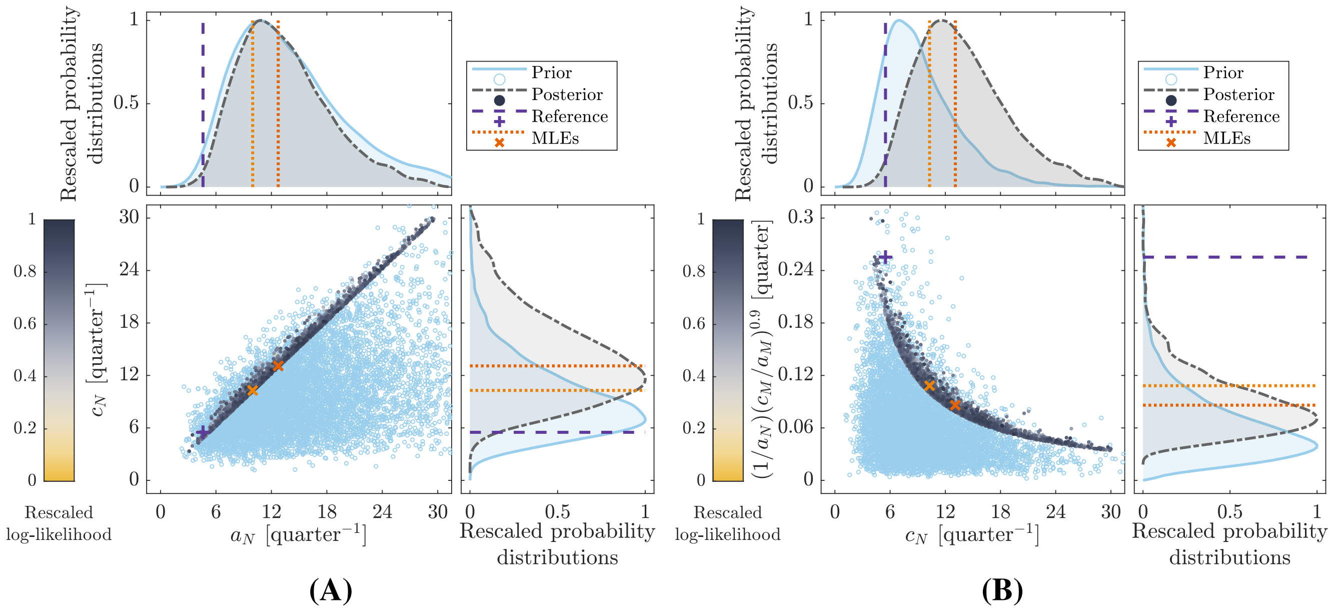

Fig. S7. Posterior distributions for the remaining stiff eigenparameters obtained from the Bayesian methods (Table 2) compared to the sets of reference values and best-fit values (MLEs). (A) $\hat{\theta}_2$ from matrix $\mathbf{P}$ and **(B)** $\hat{\theta}_2$ from matrix $\mathbf{G}$. Similar tendencies are seen for $\hat{\theta}_2$ and $\hat{\theta}_3$ from matrix $\mathbf{P}$, thus $\hat{\theta}_3$ is not shown. Many samples of the posterior distribution yield similar values of the log-likelihood function, with $a_N \propto c_N$, $a_M \propto c_M$ and $c_N \propto \left[(1/a_N)(c_M/a_M)^{0.9}\right]^{-1}$. (See also Figures' Supplementary Legends.)

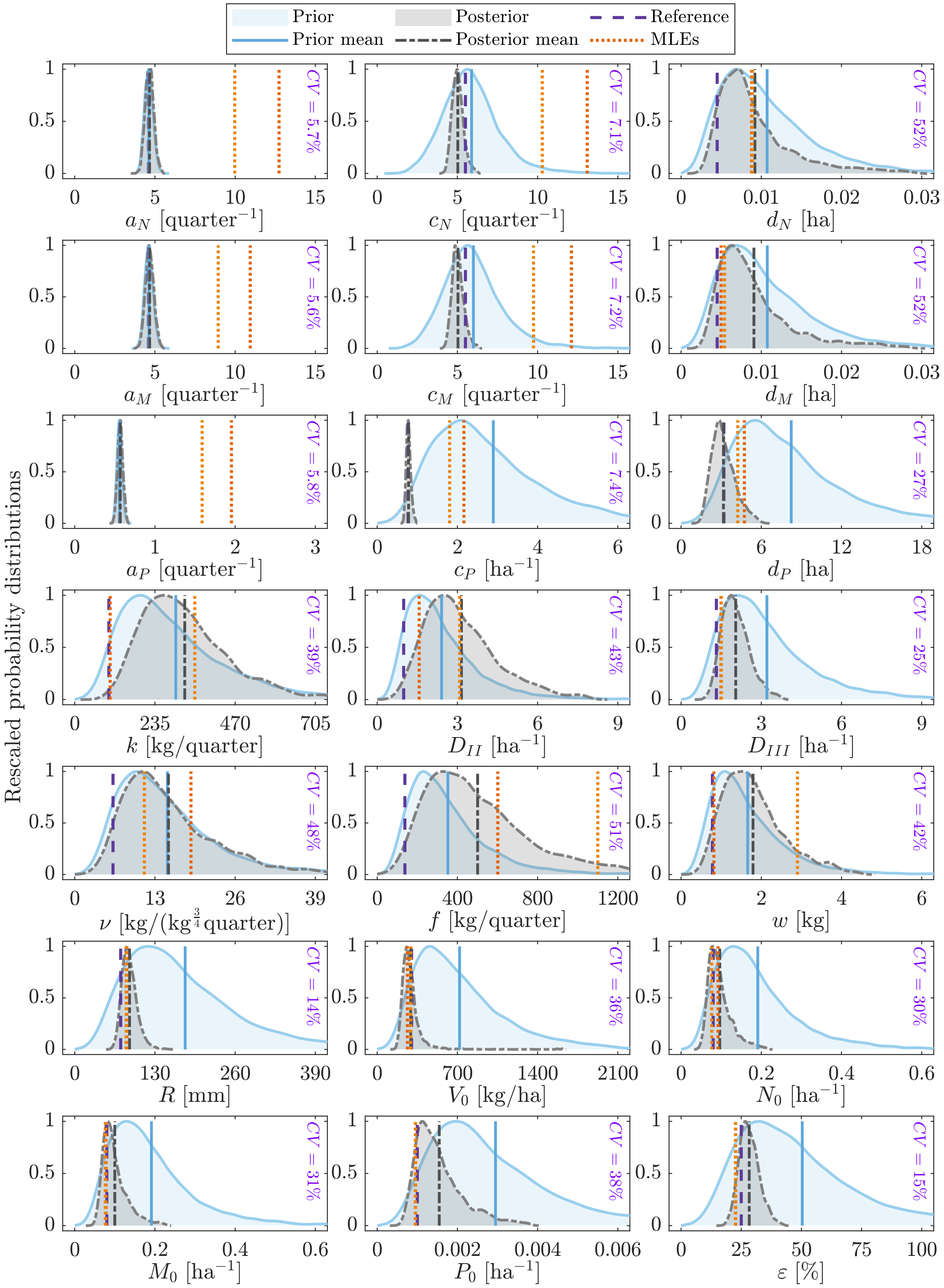

Fig. S8. Prior and posterior distributions for the parameters of the ecosystem network model (Table S1) together with the set of reference parameter values and best-fit parameter values (MLEs) considering a more informative multivariate log-normal prior for all parameters. Percentage coefficient of variation for the posterior sample of each parameter (CV) is reported within each panel (compare to Fig. S5). Improved priors for parameters a_N, a_M, a_P also constrain parameters c_N, c_M and c_P due to the identified parameter relations. (See also Figures' Supplementary Legends.)

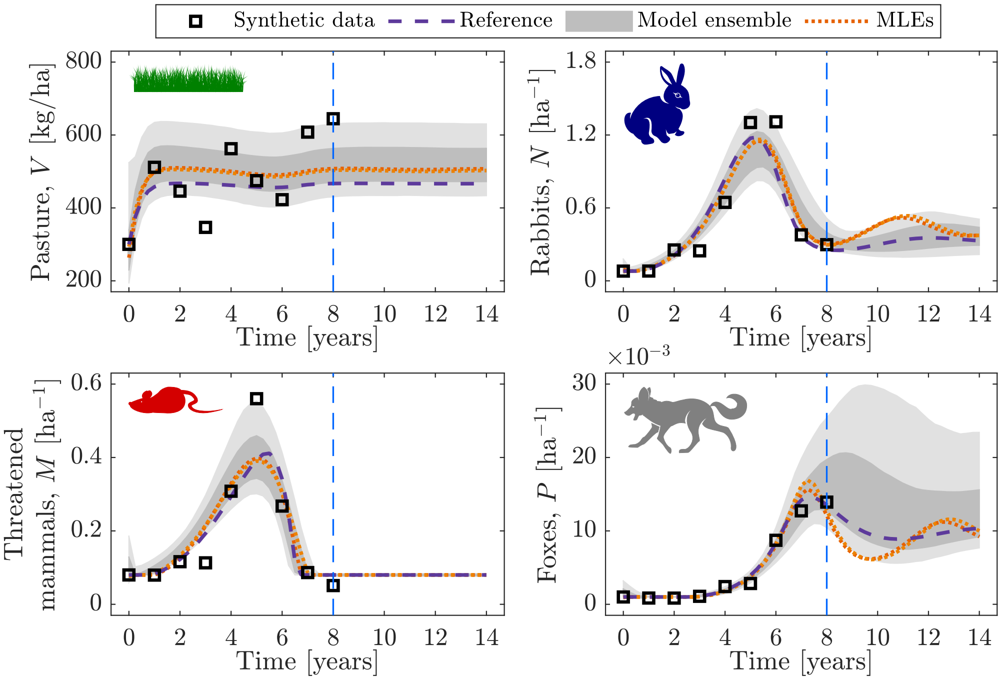

Fig. S9. Ecosystem network model fit to time-series data considering a more informative multivariate log-normal prior for all parameters in the implementation of Bayesian inference. Synthetic time-series data for ecological abundance with measurement error of $\varepsilon = 25\%$ together with noiseless model prediction using reference parameter values (Table S2), model predictions using two sets of best-fit parameter values (MLEs), and model ensemble predictions using all plausible parameter values (Fig. S8). The ecosystem network model fits the synthetic time-series data, with the model ensemble propagating parameter uncertainty into species abundance predictions. (See also Figures' Supplementary Legends.)

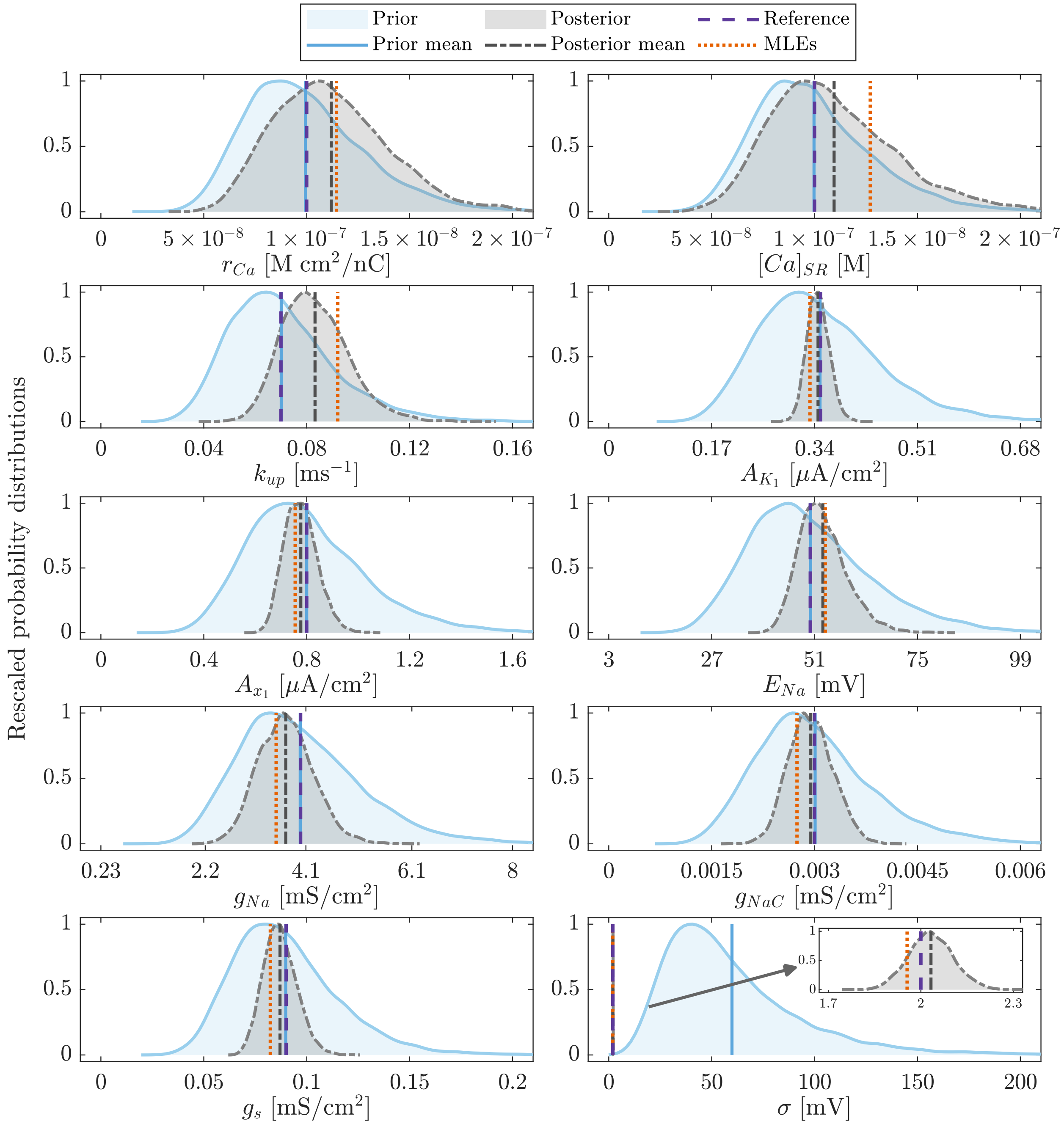

Fig. S10. Prior and posterior distributions for the parameters of the Beeler-Reuter model (Table S3) together with reference parameter values and best-fit parameter values considering a multivariate log-normal prior for all parameters. Most of the model parameters are well-constrained by the data. (See also Figures' Supplementary Legends.)

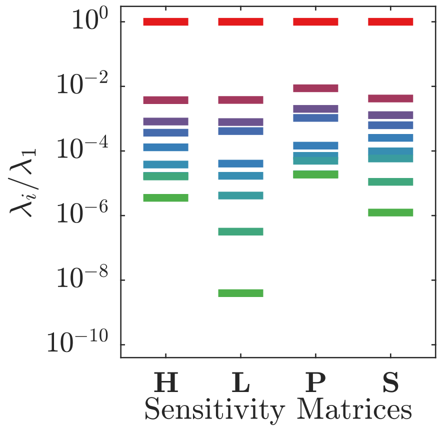

Fig. S11. Sensitivity matrix eigenvalue spectra (based on SVD) for the Beeler-Reuter model in Table S3 fit to synthetic data, considering a multivariate log-normal prior distribution for all parameters. Largest eigenvalue λ_1 is used to rescale eigenvalues between $0 - 1$.

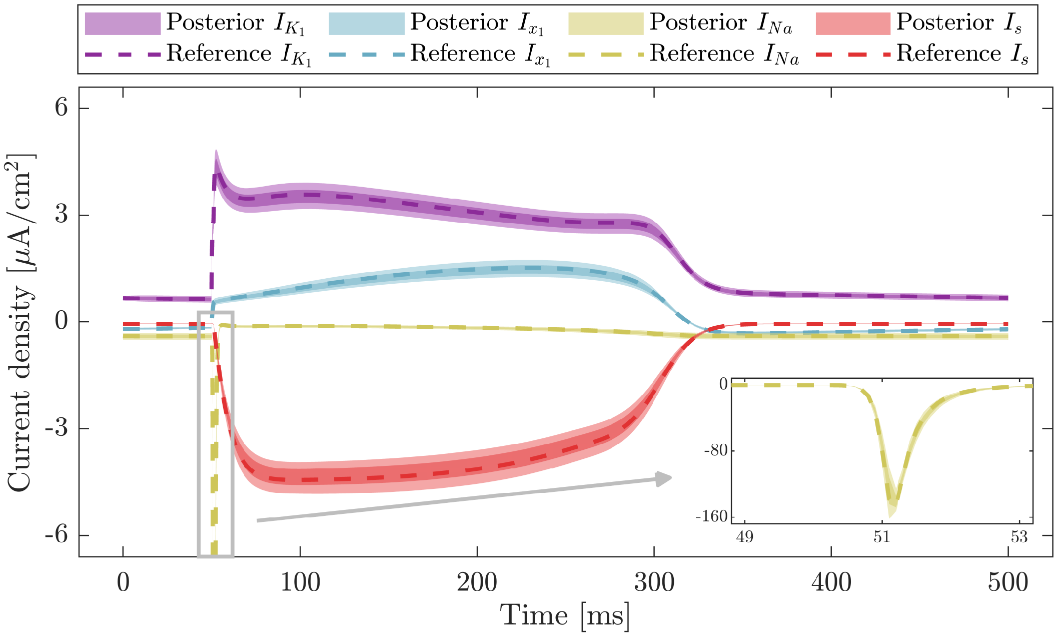

Fig. S12. Dynamics of the individual currents together composing the Beeler-Reuter model action potential. Time courses of the four currents that sum to form the action potential, displaying both those observed for the reference model (dashed lines) used to generate the synthetic data and the 68% and 95% confidence intervals (dark and light shaded regions, respectively) among the posterior population of parameter values obtained via Bayesian inference. The inset displays the sharp spike of the Na^+ current that initiates the action potential. All currents exhibit clear nonlinear behavior, and interact with each other only indirectly via the cell's overall polarization level. (See also Figures' Supplementary Legends.)

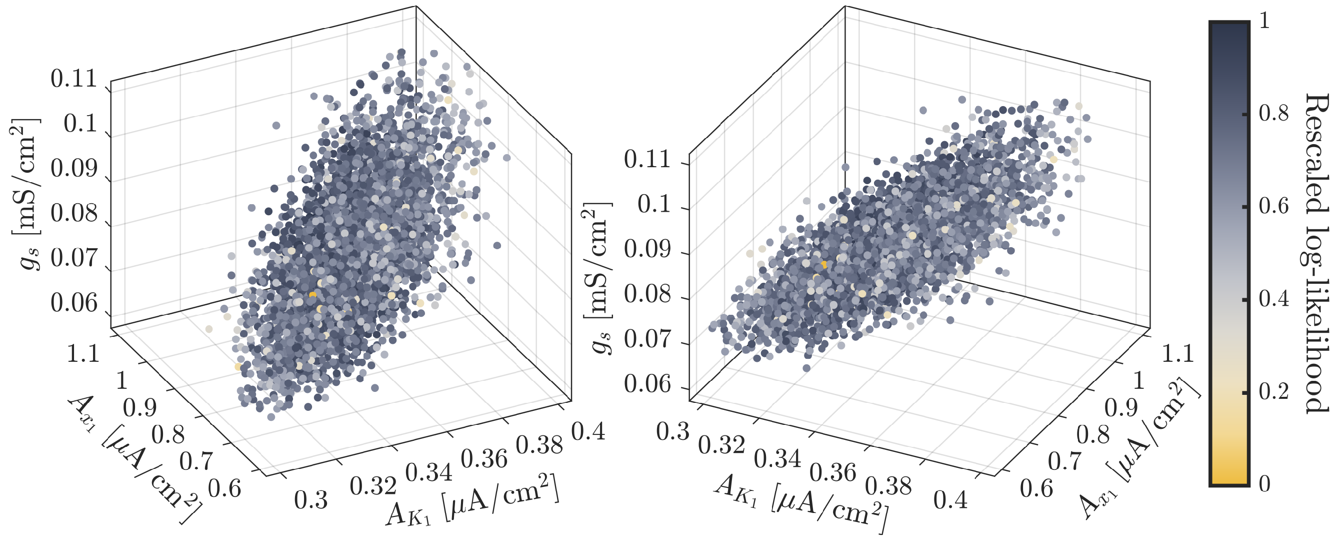

Fig. S13. Trivariate scatter plot of the posterior distribution for parameters A_{K_1}, A_{x_1} and g_s, with side view. Unlike Fig. 6B, relationship between combination of parameters A_{K_1} and A_{x_1} with parameter g_s is not easily visible from this trivariate scatter plot. (See also Figures' Supplementary Legends.)

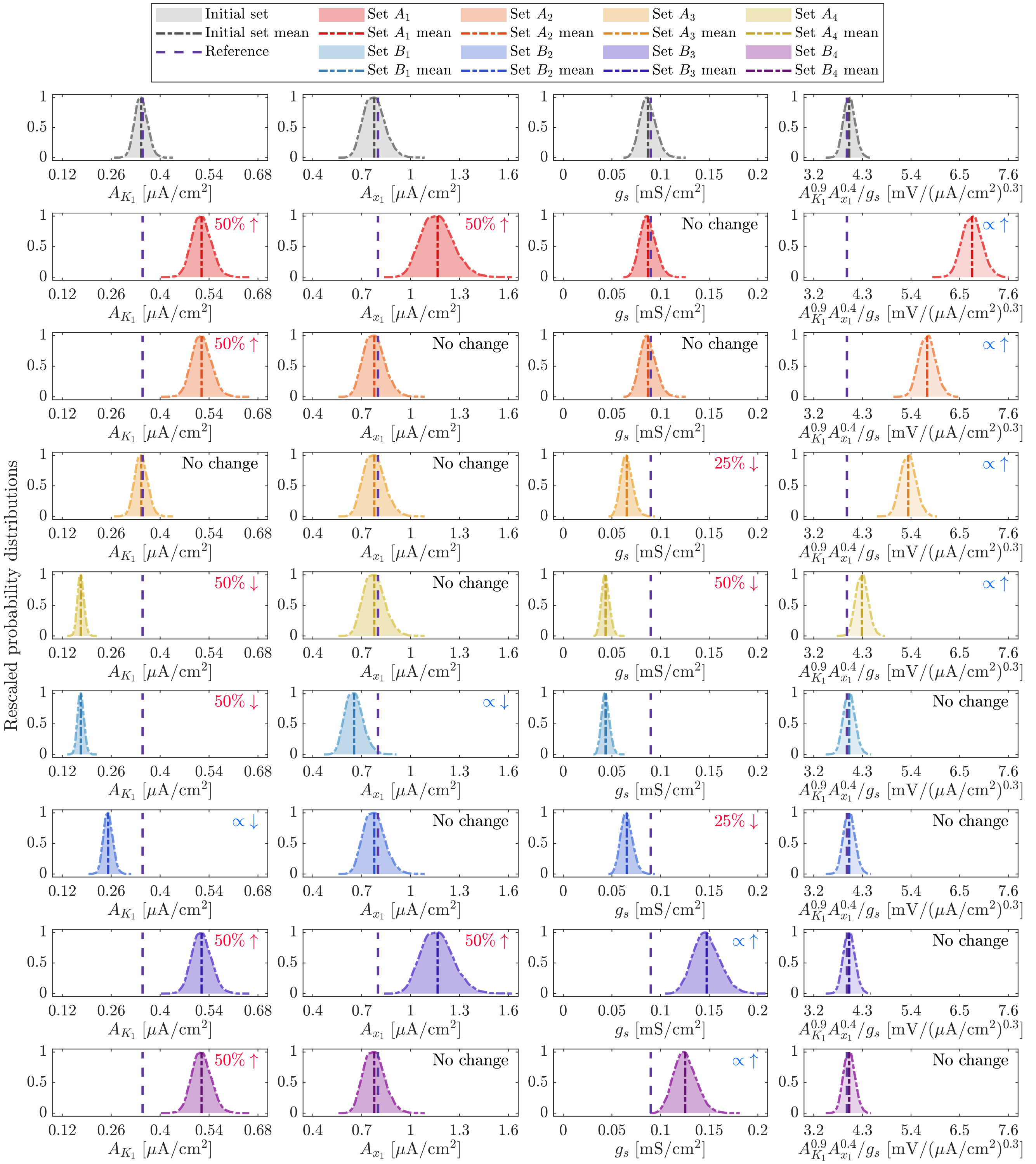

Fig. S14. Variation of parameters A_{K_1}, A_{x_1} and g_s to change or keep approximately constant the value of stiffest eigenparameter $\hat{\theta}_1 = A_{K_1}^{0.9} A_{x_1}^{0.4}/g_s$. The first row of panels depicts the initial set of parameter values, obtained via Bayesian inference while the remaining rows of panels depict eight sets of specified parameter values A_1 to A_4 and B_1 to B_4 which change or keep constant the value of the stiffest eigenparameter, respectively. Changes in the parameter values relative to their estimated values (shown in the top four panels) are indicated at the top right corner of each panel, with $\propto$ symbol indicating a proportional change in the value of the parameter or eigenparameter while ↑ and ↓ symbols indicating an increase and decrease of the parameter value, respectively. (See also Figures' Supplementary Legends.)

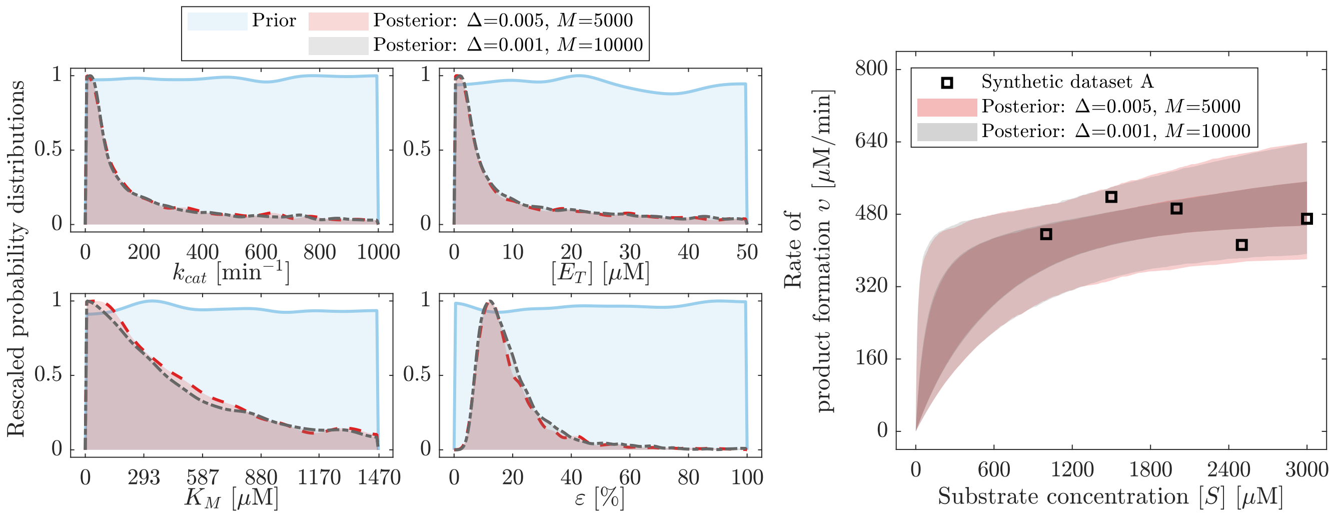

(A) **(B)**

Fig. S15. Estimated parameter values and fit of the Michaelis–Menten model to the noisy synthetic dataset A (i.e., high substrate concentration with $[S] \gg K_M$) by implementing the posterior sampling algorithm with two different combinations of the effective sample size reduction target Δ and sample size M considering uniform prior distributions for all parameters. **(A)** Prior and posterior distributions for the parameters, with the posterior distributions overlapped. **(B)** Dataset A together with the model ensemble predictions using all plausible parameter values obtained via Bayesian inference (also overlapped). Both posterior distribution and model ensemble prediction considering $\Delta = 0.005$ and $M = 5000$ reproduce those considering $\Delta = 0.001$ and $M = 10000$. (See also Figures' Supplementary Legends.)

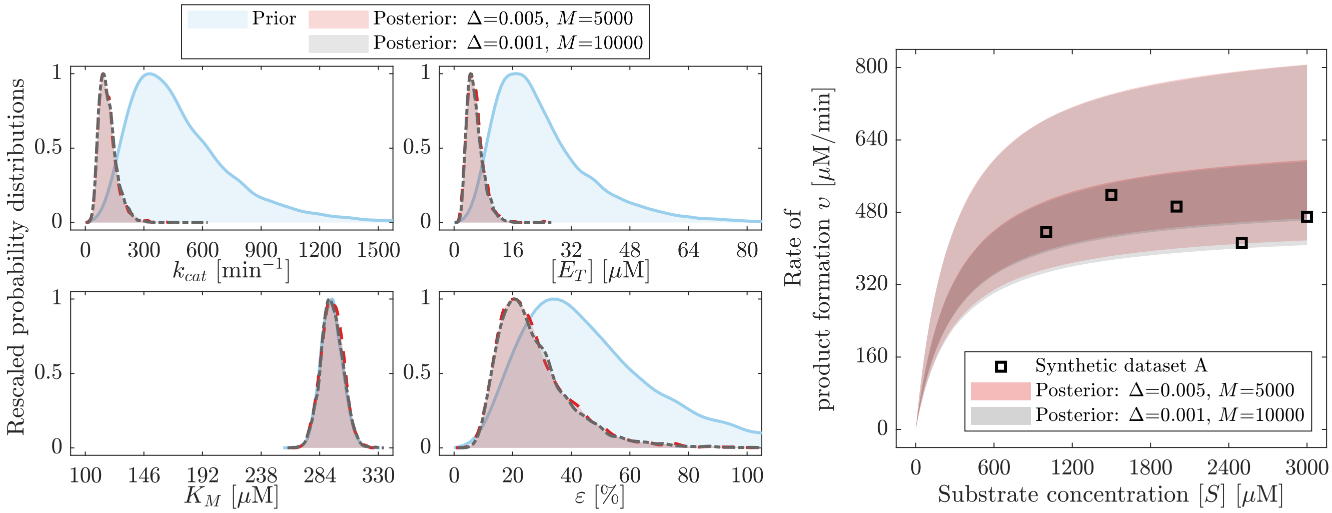

(A) **(B)**

Fig. S16. Estimated parameter values and fit of the Michaelis–Menten model to the noisy synthetic dataset A (i.e., high substrate concentration with $[S] \gg K_M$) by implementing the posterior sampling algorithm with two different combinations of the effective sample size reduction target Δ and sample size M considering a multivariate log-normal prior distribution for all parameters with that of parameter K_M badly specified with $p(K_M = 146.7\ \mu\text{M}) \approx 0$. **(A)** Prior and posterior distributions for the parameters, with the posterior distributions overlapped. **(B)** Dataset A together with the model ensemble predictions using all plausible parameter values obtained via Bayesian inference (also overlapped). Both posterior distribution and model ensemble prediction considering $\Delta = 0.005$ and $M = 5000$ reproduce those considering $\Delta = 0.001$ and $M = 10000$. (See also Figures' Supplementary Legends.)

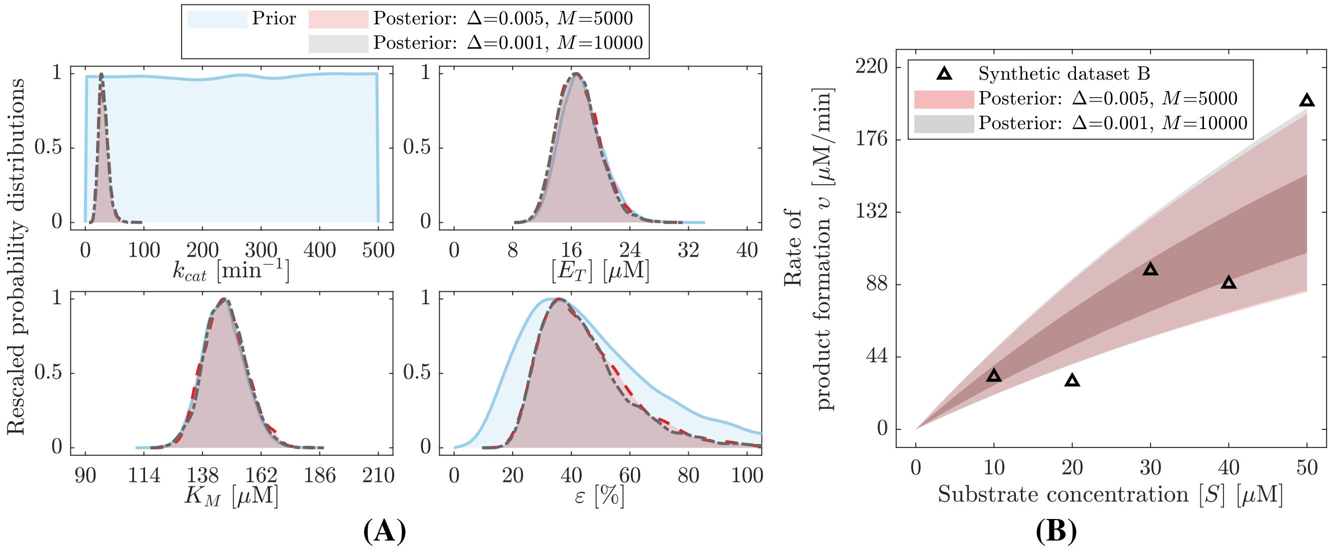

Fig. S17. Estimated parameter values and fit of the Michaelis–Menten model to the noisy synthetic dataset B (i.e., at low substrate concentration with $[S] \ll K_M$) by implementing the posterior sampling algorithm with two different combinations the effective sample size reduction target Δ and sample size M considering a uniform prior for k_{cat}, a badly specified log-normal prior for $[E_T]$ with $p([E_T] = 5\ \mu\text{M}) \approx 0$, a well-specified log-normal prior for K_M with $p(K_M = 146.7\ \mu\text{M}) \approx 1$ and a log-normal prior for σ. (A) Prior and posterior distributions for the parameters, with the posterior distributions overlapped. **(B)** Dataset B together with the model ensemble predictions using all plausible parameter values obtained via Bayesian inference (also overlapped). Both posterior distribution and model ensemble prediction considering $\Delta = 0.005$ and $M = 5000$ reproduce those considering $\Delta = 0.001$ and $M = 10000$. (See also Figures' Supplementary Legends.)

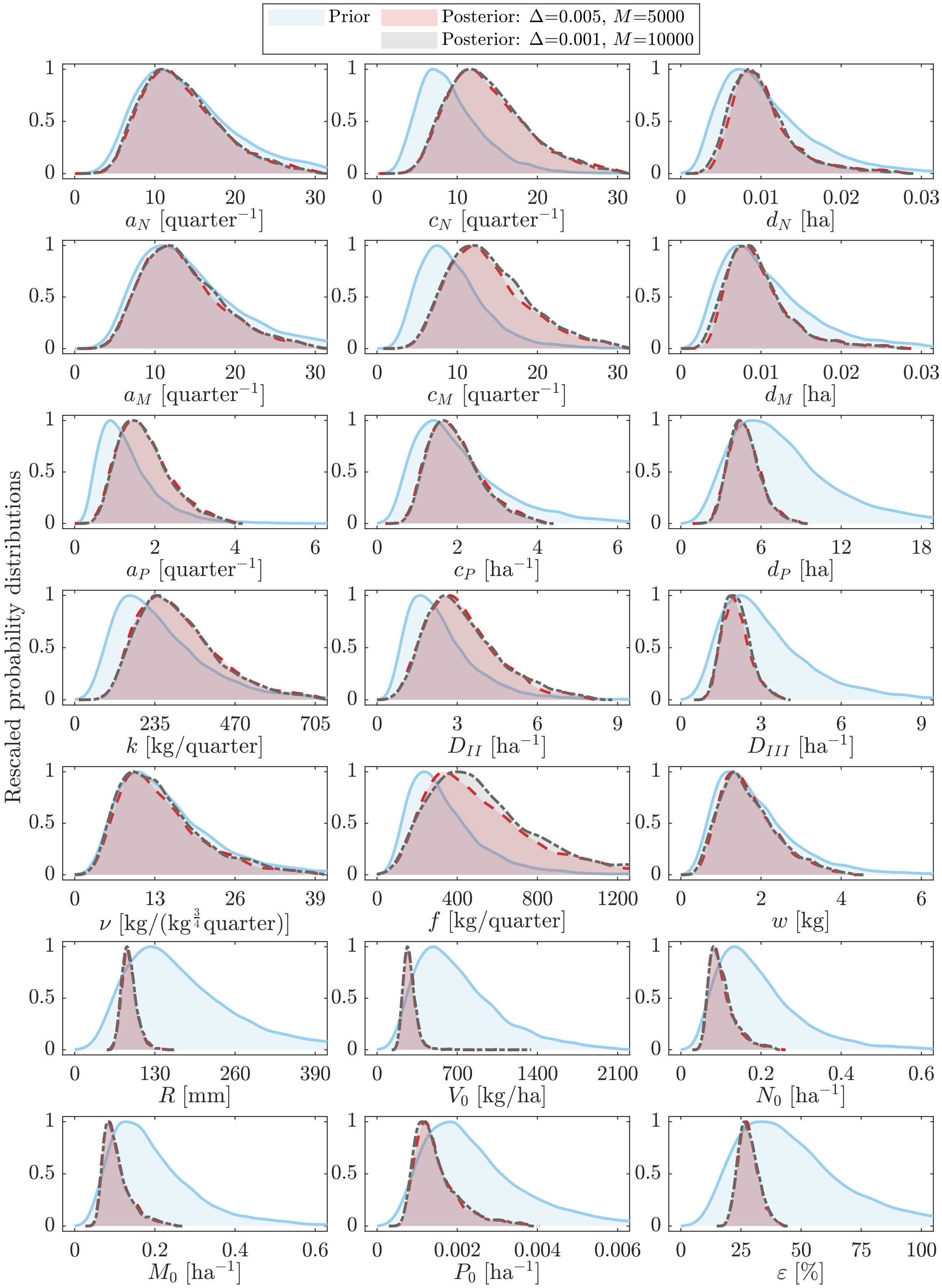

Fig. S18. Prior and posterior distributions for the parameters of the ecosystem network model (Table S1) by implementing the posterior sampling algorithm with two different combinations of the effective sample size reduction target Δ and sample size M considering a vague multivariate log-normal prior for all parameters. Posterior distribution considering $\Delta = 0.005$ and $M = 5000$ reproduces that considering $\Delta = 0.001$ and $M = 10000$. (See also Figures' Supplementary Legends.)

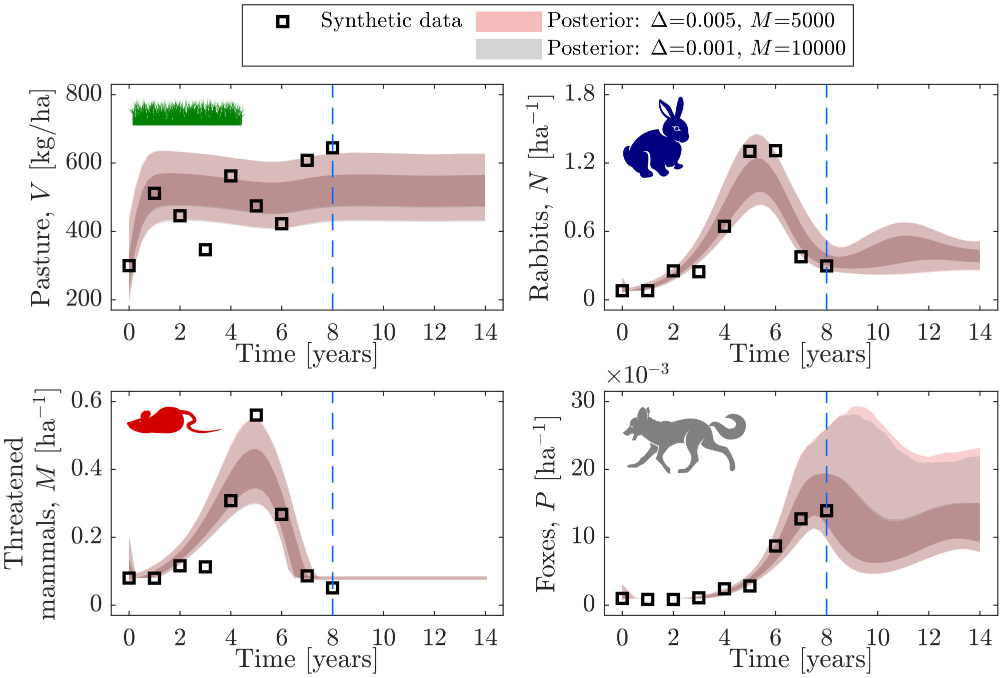

Fig. S19. Ecosystem network model fit to time-series data by implementing the posterior sampling algorithm with two different combinations of the effective sample size reduction target Δ and sample size M considering a vague multivariate log-normal prior for all parameters. Synthetic time-series data for ecological abundance with measurement error of $\varepsilon = 25\%$ together with the model ensemble predictions using all plausible parameter values (Fig. S18). Model ensemble predictions considering $\Delta = 0.005$ and $M = 5000$ reproduce those considering $\Delta = 0.001$ and $M = 10000$. (See also Figures' Supplementary Legends.)

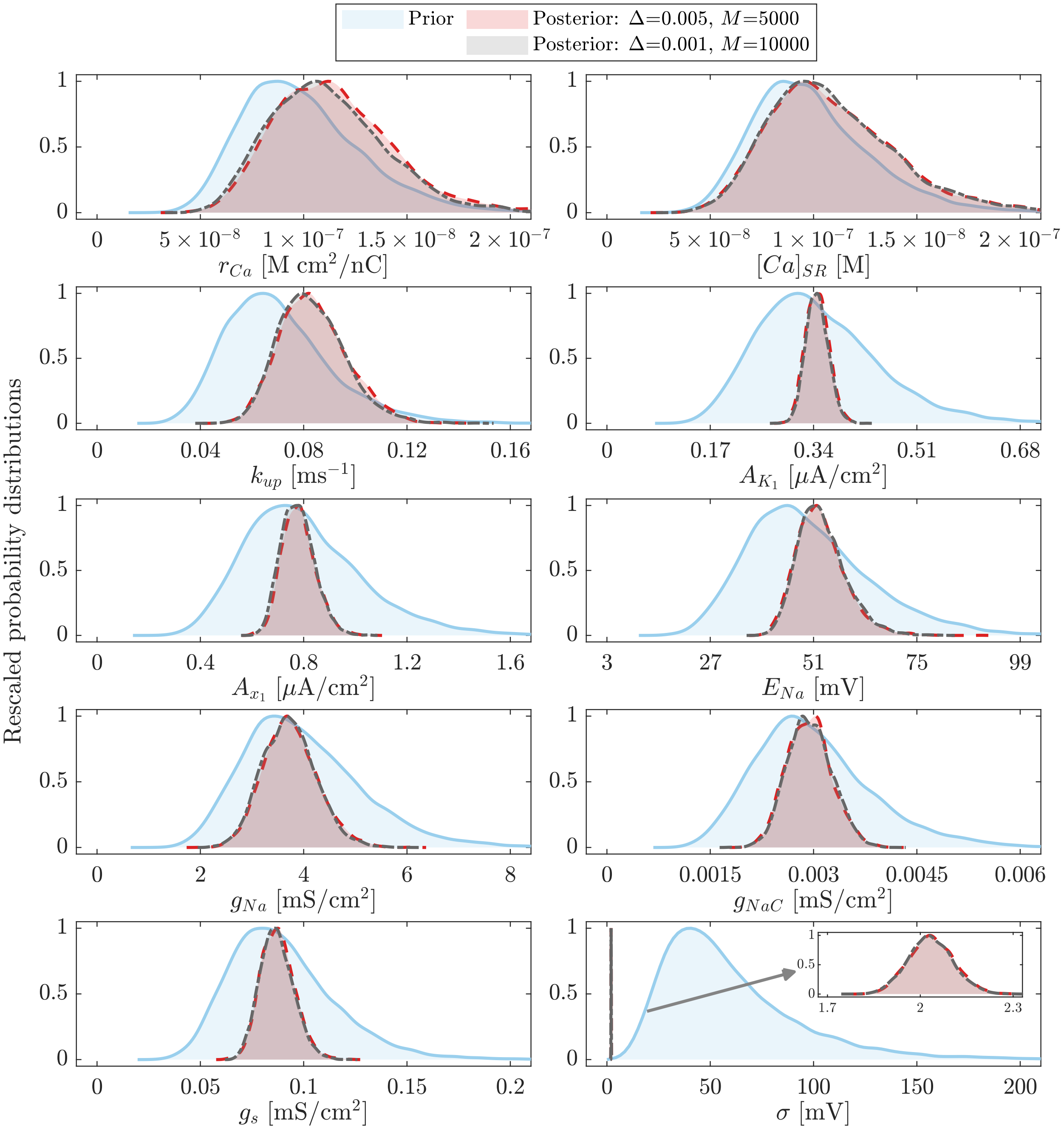

Fig. S20. Prior and posterior distributions for the parameters of the Beeler-Reuter model (Table S3) by implementing the posterior sampling algorithm with two different combinations of the effective sample size reduction target Δ and sample size M considering a multivariate log-normal prior for all parameters. Posterior distribution considering $\Delta = 0.005$ and $M = 5000$ reproduces that considering $\Delta = 0.001$ and $M = 10000$. (See also Figures' Supplementary Legends.)

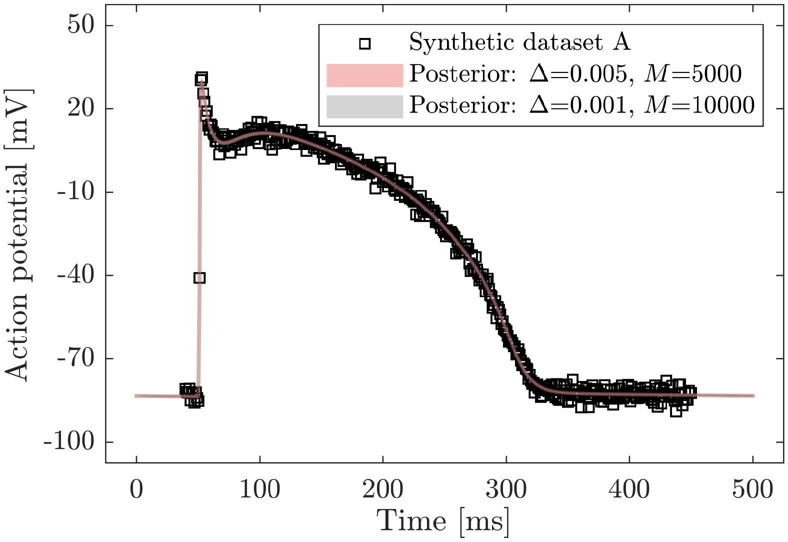

Fig. S21. Beeler-Reuter model fit to synthetic time-series data by implementing the posterior sampling algorithm with two different combinations of the effective sample size reduction target Δ and sample size M considering a multivariate log-normal prior for all parameters. Synthetic action potential (AP) data with measurement error of $\sigma = 2\text{mV}$ and time resolution of 1 ms (1 kHz) together with the model ensemble predictions using all plausible parameter values (Fig. S20). Model ensemble predictions considering $\Delta = 0.005$ and $M = 5000$ reproduce those considering $\Delta = 0.001$ and $M = 10000$. (See also Figures' Supplementary Legends.)

Equation		Description
**Pasture biomass (V):**		
$\frac{\Delta V}{\Delta t} = r_V(t) - g_N(t)N - h_M(t)M$	(S.1.1)	Pasture biomass at time t
$r_V(t) = -55.12 - 0.0153V - 0.00056V^2 + 2.5R$	(S.1.2)	Pasture biomass growth rate
$g_N(t) = h_M(t) = \nu w^{3/4}\left[1 - \exp\left\{-\frac{V + r_V(t)\Delta t}{f}\right\}\right]$	(S.1.3)	Pasture consumed by rabbits & threatened species
**Rabbit density (N):**		
$\frac{\Delta N}{\Delta t} = r_N(t)N - g_P(t)P, \quad N \geq 0.08\ \mathrm{ha}^{-1}$	(S.1.4)	Rabbit density at time t
$r_N(t) = -a_N + c_N\left[1 - \exp\left\{-d_N V\right\}\right]$	(S.1.5)	Numerical response of rabbits
$g_P(t) = \frac{[k/w]N^2}{N^2 + D_{III}^2}$	(S.1.6)	Functional response of foxes to rabbits
**Threatened species density (M):**		
$\frac{\Delta M}{\Delta t} = r_M(t)M - q_P(t)P, \quad M \geq 0.08\ \mathrm{ha}^{-1}$	(S.1.7)	Threatened species density at time t
$r_M(t) = -a_M + c_M\left[1 - \exp\left\{-d_M V\right\}\right]$	(S.1.8)	Numerical response of threatened species
$q_P(t) = h_P\left[1 - \frac{g_P(t)}{k}\right]$	(S.1.9)	Fox predation rate on threatened species
$h_P = \frac{[k/w]M}{M + D_{II}}$	(S.1.10)	Functional response of foxes to threatened species
**Fox density (P):**		
$\frac{\Delta P}{\Delta t} = r_P(t)P, \quad P \geq 0.001\mathrm{ha}^{-1}$	(S.1.11)	Fox density at time t
$r_P(t) = -a_P + c_P\left[1 - \exp\left\{-d_P(M + P)\right\}\right]$	(S.1.12)	Numerical response of foxes

Table S1. Equations of the ecosystem network model. Model developed by Pech and Hood (*42*).

**Symbol**	$\boldsymbol{\theta_R}$	**Units**	**Description**
a_N	4.60	quarter^{-1}	Maximum rate of decrease of rabbit population in absence of food
c_N	5.50	quarter^{-1}	Maximum rate of increase of rabbit population when food is abundant
d_N	0.0045	ha	Demographic efficiency of rabbit population
a_M	4.60	quarter^{-1}	Maximum rate of decrease of threatened species population in absence of food
c_M	5.50	quarter^{-1}	Maximum rate of increase of threatened species population when food is abundant
d_M	0.0045	ha	Demographic efficiency of threatened species population
a_P	0.56	quarter^{-1}	Maximum rate of decrease of fox population in absence of food
c_P	0.77	quarter^{-1}	Maximum rate of increase of fox population when food is abundant
d_P	3.20	ha	Demographic efficiency of fox population
k	100.01	kg/quarter	Maximum food consumption rate of fox population
D_{II}	0.99	ha^{-1}	Limiting fox consumption rate when rabbit population is low
D_{III}	1.32	ha^{-1}	Density of rabbits at the inflection point
ν	6.21	kg/(kg$^{3/4}$quarter)	Maximum (satiation) body-weight adjusted consumption rate per animal
f	138.00	kg/ha	Biomass at which the herbivore's intake is depressed to 63% of $\nu w^{3/4}$
w	0.78	kg	Mean body weight of rabbits eaten by foxes
$R^\dagger$	74.48	mm	Quarterly-averaged rainfall
V_0	300.00	kg/ha	Initial pasture biomass
N_0	0.080	ha^{-1}	Initial rabbit population
M_0	0.080	ha^{-1}	Initial threatened species population
P_0	0.001	ha^{-1}	Initial fox population

Table S2. Reference parameter values $\boldsymbol{\theta_R}$ for the ecosystem network model. Parameter values reported by Pech and Hood (*42*). †Parameter R is calculated as the mean of the quarterly rainfall statistics for Lerida reported by Pech and Hood (*42*).

Equation		Description
**Membrane potential (V_m):**		
$\frac{\mathrm{d}V_m}{\mathrm{d}t} = -\frac{1}{C_m}\left[i_{K_1} + i_{x_1} + i_{Na} + i_s - i_{stim}\right]$	(S.2.1)	Dynamics of the membrane potential
$i_{K_1} = A_{K_1}\left[\frac{4(\exp\{0.04(V_m+85)\}-1)}{\exp\{0.08(V_m+53)\}+\exp\{0.04(V_m+53)\}} + \frac{0.2(V_m+23)}{1-\exp\{-0.04(V_m+23)\}}\right]$	(S.2.2)	Time-independent outward potassium ion (K^+) current
$i_{x_1} = A_{x_1}x_1\left[\frac{\exp\{0.04(V_m+77\}-1}{\exp\{0.04(V_m+35)\}}\right]$	(S.2.3)	Time- and voltage-dependent outward potassium ion (K^+) current
$i_{Na} = \left(g_{Na}m^3hj + g_{NaC}\right)(V_m - E_{Na})$	(S.2.4)	Two inward sodium ion (Na^+) current (fast and background)
$i_s = g_s df\,(V_m - E_s)$	(S.2.5)	Slow inward calcium ion (Ca^{2+}) current
$i_{stim} = \begin{cases} A_s & t_{on} \leq t < t_{on} + t_{dur} \\ 0 & \text{otherwise} \end{cases}$	(S.2.6)	Applied stimulus current
**Intracellular calcium ion concentration ($[Ca]_i$):**		
$\frac{\mathrm{d}[Ca]_i}{\mathrm{d}t} = -r_{Ca}i_s + k_{up}\left([Ca]_{SR-[Ca]_i}\right)$	(S.2.7)	Dynamics of intracellular calcium ion (Ca^{2+}) concentration
$E_s = -82.3 - 13.0287\ln[Ca]_i$	(S.2.8)	Equilibrium (Nernst) potential of calcium ion (Ca^{2+})
**Ion channel gating variables (x_1, m, h, j, d and f):**		
$\frac{\mathrm{d}x_1}{\mathrm{d}t} = (1-x_1)\left[\frac{0.0005\exp\{0.083(V_m+50)\}}{\exp\{0.057(V_m+50)\}+1}\right] - x_1\left[\frac{0.0013\exp\{-0.06(V_m+20)\}}{\exp\{-0.04(V_m+20)\}+1}\right]$	(S.2.9)	Dynamics of potassium ion (K^+) activation gate
$\frac{\mathrm{d}m}{\mathrm{d}t} = (1-m)\left[\frac{-(V_m+47)}{\exp\{-0.1(V_m+47)\}-1}\right] - m\left[40\exp\{-0.056(V_m+72)\}\right]$	(S.2.10)	Dynamics of sodium ion (Na^+) activation gate
$\frac{\mathrm{d}h}{\mathrm{d}t} = (1-h)\left[0.126\exp\{-0.25(V_m+77)\}\right] - h\left[\frac{1.7}{\exp\{-0.082(V_m+22.5)\}+1}\right]$	(S.2.11)	Dynamics of sodium ion (Na^+) fast inactivation gate
$\frac{\mathrm{d}j}{\mathrm{d}t} = (1-j)\left[\frac{0.055\exp\{-0.25(V_m+78)\}}{\exp\{-0.2(V_m+78)\}+1}\right] - j\left[\frac{0.3}{\exp\{-0.1(V_m+32)\}+1}\right]$	(S.2.12)	Dynamics of sodium ion (Na^+) slow inactivation gate
$\frac{\mathrm{d}d}{\mathrm{d}t} = (1-d)\left[\frac{0.095\exp\{-0.01(V_m-5)\}}{\exp\{-0.072(V_m-5)\}+1}\right] - d\left[\frac{0.07\exp\{-0.017(V_m+44)\}}{\exp\{0.05(V_m+44)\}+1}\right]$	(S.2.13)	Dynamics of calcium ion (Ca^{2+}) activation gate
$\frac{\mathrm{d}f}{\mathrm{d}t} = (1-f)\left[\frac{0.012\exp\{-0.008(V_m+28)\}}{\exp\{0.15(V_m+28)\}+1}\right] - f\left[\frac{0.065\exp\{-0.02(V_m+30)\}}{\exp\{-0.2(V_m+30)\}+1}\right]$	(S.2.14)	Dynamics of calcium ion (Ca^{2+}) inactivation gate

Table S3. Equations of the cardiac action potential (AP) model (*88*). Model developed by Beeler and Reuter (*43*).

	Symbol	$\boldsymbol{\theta_R}$	Units	Description
**Estimated model parameters**	r_{Ca}	1×10^{-7}	M $\mathrm{cm}^2/\mathrm{nC}$	Calcium ion (Ca^{2+}) intracellular uptake rate
	$[Ca]_{SR}$	1×10^{-7}	M	Free calcium ion (Ca^{2+}) concentration in the sarcoplasmic reticulum (SR)
	k_{up}	0.07	ms^{-1}	Calcium ion (Ca^{2+}) uptake rate by the sarcoplasmic reticulum
	A_{K_1}	0.35	$\mu\mathrm{A/cm}^2$	Maximal current density of time-independent outward potassium ion (K^+) current
	A_{x_1}	0.8	$\mu\mathrm{A/cm}^2$	Maximal current density of time- and voltage-dependent outward potassium ion (K^+) current
	E_{Na}	50	mV	Equilibrium potential of sodium ion (Na^+)
	g_{Na}	4	$\mathrm{mS/cm}^2$	Conductance of background inward sodium ion (Na^+) current
	g_{NaC}	0.003	$\mathrm{mS/cm}^2$	Conductance of fast inward sodium ion (Na^+) current
	g_s	0.09	$\mathrm{mS/cm}^2$	Conductance of slow inward calcium ion (Ca^{2+}) current
**Fixed model parameters**	A_s	40	$\mu\mathrm{A/cm}^2$	Stimulus current
	t_{on}	50	ms	Stimulus start time
	t_{dur}	1	ms	Stimulus duration
	C_m	1	$\mu\mathrm{F/cm}^2$	Membrane capacitance
	$V_m(0)$	-83.3	mV	Initial membrane potential
	$[Ca]_i(0)$	1.87×10^{-7}	M	Initial intracellular calcium ion (Ca^{2+}) concentration
	$x_1(0)$	0.1644	Unitless	Initial value of the potassium ion (K^+) activation gate
	$m(0)$	0.01	Unitless	Initial value of the sodium ion (Na^+) activation gate
	$h(0)$	0.9814	Unitless	Initial value of the sodium ion (Na^+) fast inactivation gate
	$j(0)$	0.9673	Unitless	Initial value of the sodium ion (Na^+) slow inactivation gate
	$d(0)$	0.0033	Unitless	Initial value of the calcium ion (Ca^{2+}) activation gate
	$f(0)$	0.9884	Unitless	Initial value of the calcium ion (Ca^{2+}) inactivation gate

Table S4. Reference (true) parameter values $\boldsymbol{\theta_R}$ for the cardiac action potential (AP) model (*88*). Parameter values originally reported by Beeler and Reuter (*43*). Estimated model parameters correspond to those whose values are estimated in this work via Bayesian inference or maximum likelihood estimation. Fixed model parameters are set to their reference value, and thus they are not estimated via our model-data fitting techniques.

REFERENCES AND NOTES

1. A. Ma'ayan, Complex systems biology. *J. R. Soc. Interface* **14**, 20170391 (2017).

2. W. L. Geary, M. Bode, T. S. Doherty, E. A. Fulton, D. G. Nimmo, A. I. T. Tulloch, V. J. D. Tulloch, E. G. Ritchie, A guide to ecosystem models and their environmental applications. *Nat. Ecol. Evol.* **4**, 1459–1471 (2020).

3. A. F. Villaverde, J. R. Banga, Reverse engineering and identification in systems biology: Strategies, perspectives and challenges. *J. R. Soc. Interface* **11**, 20130505 (2014).

4. N. Mouquet, Y. Lagadeuc, V. Devictor, L. Doyen, A. Duputié, D. Eveillard, D. Faure, E. Garnier, O. Gimenez, P. Huneman, F. Jabot, P. Jarne, D. Joly, R. Julliard, S. Kéfi, G. J. Kergoat, S. Lavorel, L. L. Gall, L. Meslin, S. Morand, X. Morin, H. Morlon, G. Pinay, R. Pradel, F. M. Schurr, W. Thuiller, M. Loreau, REVIEW: Predictive ecology in a changing world. *J. Appl. Ecol.* **52**, 1293–1310 (2015).

5. C. C. Drovandi, A. N. Pettitt, Estimation of parameters for macroparasite population evolution using approximate Bayesian computation. *Biometrics* **67**, 225–233 (2011).

6. T. Schlick, *Molecular Modeling and Simulation: An Interdisciplinary Guide* (Springer, 2010), vol. 2.

7. B. A. J. Lawson, C. C. Drovandi, N. Cusimano, P. Burrage, B. Rodriguez, K. Burrage, Unlocking data sets by calibrating populations of models to data density: A study in atrial electrophysiology. *Sci. Adv.* **4**, e1701676 (2018).

8. R. H. Johnstone, E. T. Y. Chang, R. Bardenet, T. P. de Boer, D. J. Gavaghan, P. Pathmanathan, R. H. Clayton, G. R. Mirams, Uncertainty and variability in models of the cardiac action potential: Can we build trustworthy models? *J. Mol. Cell. Cardiol.* **96**, 49–62 (2016).

9. K. Velten, *Mathematical Modeling and Simulation: Introduction for Scientists and Engineers* (Wiley-VCH, 2009).

10. M. Sundberg, Creating convincing simulations in astrophysics. *Sci. Technol. Human Values* **37**, 64–87 (2010).

11. R. N. Gutenkunst, J. J. Waterfall, F. P. Casey, K. S. Brown, C. R. Myers, J. P. Sethna, Universally sloppy parameter sensitivities in systems biology models. *PLOS Comput. Biol.* **3**, e189 (2007).

12. K. S. Brown, J. P. Sethna, Statistical mechanical approaches to models with many poorly known parameters. *Phys. Rev. E* **68**, 021904 (2003).

13. K. S. Brown, C. C. Hill, G. A. Calero, C. R. Myers, K. H. Lee, J. P. Sethna, R. A. Cerione, The statistical mechanics of complex signaling networks: Nerve growth factor signaling. *Phys. Biol.* **1**, 184 (2004).

14. A. Lewbel, The identification zoo: Meanings of identification in econometrics. *J. Econ. Lit.* **57**, 835–903 (2019).

15. L. Geris, D. Gomez-Cabrero, *Uncertainty in Biology: A Computational Modeling Approach* (Springer International Publishing, 2016).

16. M. K. Transtrum, B. B. Machta, J. P. Sethna, Geometry of nonlinear least squares with applications to sloppy models and optimization. *Phys. Rev. E* **83**, 036701 (2011).

17. M. P. Adams, S. A. Sisson, K. J. Helmstedt, C. M. Baker, M. H. Holden, M. Plein, J. Holloway, K. L. Mengersen, E. McDonald-Madden, Informing management decisions for ecological networks, using dynamic models calibrated to noisy time-series data. *Ecol. Lett.* **23**, 607–619 (2020).

18. S. Marino, I. B. Hogue, C. J. Ray, D. E. Kirschner, A methodology for performing global uncertainty and sensitivity analysis in systems biology. *J. Theor. Biol.* **254**, 178–196 (2008).

19. A. Saltelli, T. H. Andres, T. Homma, Sensitivity analysis of model output: An investigation of new techniques. *Comput. Stat. Data Anal.* **15**, 211–238 (1993).

20. I. M. Sobol', Global sensitivity indices for nonlinear mathematical models and their Monte Carlo estimates. *Math. Comput. Simul.* **55**, 271–280 (2001).

21. A. Saltelli, M. Ratto, T. Andres, F. Campolongo, J. Cariboni, D. Gatelli, M. Saisana, S. Tarantola, *Global Sensitivity Analysis: The Primer* (John Wiley & Sons Ltd, 2008).

22. M. Girolami, Bayesian inference for differential equations. *Theor. Comput. Sci.* **408**, 4–16 (2008).

23. A. Gelman, J. B. Carlin, H. S. Stern, D. B. Dunson, A. Vehtari, D. B. Rubin, *Bayesian Data Analysis* (Chapman and Hall/CRC, ed. 3, 2013).

24. D. Luengo, L. Martino, M. Bugallo, V. Elvira, S. Särkkä, A survey of Monte Carlo methods for parameter estimation. *EURASIP J. Adv. Signal Process.* **2020**, 25 (2020).

25. C. C. Drovandi, N. Cusimano, S. Psaltis, B. A. J. Lawson, A. N. Pettitt, P. Burrage, K. Burrage, Sampling methods for exploring between-subject variability in cardiac electrophysiology experiments. *J. R. Soc. Interface* **13**, 20160214 (2016).

26. M. K. Transtrum, B. B. Machta, K. S. Brown, B. C. Daniels, C. R. Myers, J. P. Sethna, Perspective: Sloppiness and emergent theories in physics, biology, and beyond. *J. Chem. Phys.* **143**, 010901 (2015).

27. A. White, M. Tolman, H. D. Thames, H. R. Withers, K. A. Mason, M. K. Transtrum, The limitations of model-based experimental design and parameter estimation in sloppy systems. *PLOS Comput. Biol.* **12**, e1005227 (2016).

28. M. K. Transtrum, A. T. Sarić, A. M. Stanković, Measurement-directed reduction of dynamic models in power systems. *IEEE Trans. Power Syst.* **32**, 2243 (2016).

29. D. R. Hagen, J. K. White, B. Tidor, Convergence in parameters and predictions using computational experimental design. *Interface Focus* **3**, 20130008 (2013).

30. J. F. Apgar, D. K. Witmer, F. M. White, B. Tidor, Sloppy models, parameter uncertainty, and the role of experimental design. *Mol. Biosyst.* **6**, 1890–1900 (2010).

31. E. Dufresne, H. A. Harrington, D. V. Raman, The geometry of sloppiness. *J. Algebraic Stat.* **9**, 30–68 (2018).

32. P. P.-Y. Wu, M. J. Caley, G. A. Kendrick, K. McMahon, K. Mengersen, Dynamic Bayesian network inferencing for non-homogeneous complex systems. *Appl. Stat.* **67**, 417–434 (2018).

33. S. L. Choy, R. O'Leary, K. Mengersen, Elicitation by design in ecology: Using expert opinion to inform priors for Bayesian statistical models. *Ecology* **90**, 265–277 (2009).

34. C. M. Baker, M. Bode, N. Dexter, D. B. Lindenmayer, C. Foster, C. M. Gregor, M. Plein, E. McDonald-Madden, A novel approach to assessing the ecosystem-wide impacts of reintroductions. *Ecol. Appl.* **29**, e01811 (2019).

35. T. Cui, J. Martin, Y. M. Marzouk, A. Solonen, A. Spantini, Likelihood-informed dimension reduction for nonlinear inverse problems. *Inverse Probl.* **30**, 114015 (2014).

36. H. Hotelling, Analysis of a complex of statistical variables into principal components. *J. Educ. Psychol.* **24**, 417–441 (1933).

37. A. Spantini, A. Solonen, T. Cui, J. Martin, L. Tenorio, Y. Marzouk, Optimal low-rank approximations of Bayesian linear inverse problems. *SIAM J. Sci. Comput.* **37**, A2451–A2487 (2015).

38. I. T. Jolliffe, J. Cadima, Principal component analysis: A review and recent developments. *Philos. Trans. R. Soc. A* **374**, 20150202 (2016).

39. T. J. Rothenberg, Identification in parametric models. *Econometrica* **39**, 577–591 (1971).

40. F. P. Casey, D. Baird, Q. Feng, R. N. Gutenkunst, J. J. Waterfall, C. R. Myers, K. S. Brown, R. A. Cerione, J. P. Sethna, Optimal experimental design in an epidermal growth factor receptor signalling and down-regulation model. *IET Syst. Biol.* **1**, 190–202 (2007).

41. L. Michaelis, M. Menten, Die Kinetik der Invertinwirkung. *Biochem. Z* **49**, 333 (1913).

42. R. P. Pech, G. M. Hood, Foxes, rabbits, alternative prey and rabbit calicivirus disease: Consequences of a new biological control agent for an outbreaking species in Australia. *J. Appl. Ecol.* **35**, 434–453 (1998).

43. G. W. Beeler, H. Reuter, Reconstruction of the action potential of ventricular myocardial fibres. *J. Physiol.* **268**, 177–210 (1977).

44. F.-G. Wieland, A. L. Hauber, M. Rosenblatt, C. Tönsing, J. Timmer, On structural and practical identifiability. *Curr. Opin. Syst. Biol.* **25**, 60–69 (2021).

45. B. Choi, G. A. Rempala, J. K. Kim, Beyond the Michaelis-Menten equation: Accurate and efficient estimation of enzyme kinetic parameters. *Sci. Rep.* **7**, 17018 (2017).

46. G. E. Briggs, J. B. Haldane, A note on the kinetics of enzyme action. *Biochem. J.* **19**, 338–339 (1925).

47. J. M. Tomczak, E. Węglarz-Tomczak, Estimating kinetic constants in the Michaelis-Menten model from one enzymatic assay using Approximate Bayesian Computation. *FEBS Lett.* **593**, 2742–2750 (2019).

48. T. Cui, Y. Marzouk, K. Willcox, Scalable posterior approximations for large-scale Bayesian inverse problems via likelihood-informed parameter and state reduction. *J. Comput. Phys.* **315**, 363–387 (2016).

49. T. Krogh-Madsen, D. J. Christini, *Modeling and Simulating Cardiac Electrical Activity* (IOP Publishing, 2020).

50. X. Zhou, A. Bueno-Orovio, B. Rodriguez, In silico evaluation of arrhythmia. *Curr. Opin. Phys.* **1**, 95–103 (2018).

51. O. J. Britton, A. Bueno-Orovio, K. Van Ammel, H. R. Lu, R. Towart, D. J. Gallacher, B. Rodriguez, Experimentally calibrated population of models predicts and explains intersubject

variability in cardiac cellular electrophysiology. *Proc. Natl. Acad. Sci. U.S.A.* **110**, E2098–E2105 (2013).

52. E. Passini, O. J. Britton, H. R. Lu, J. Rohrbacher, A. N. Hermans, D. J. Gallacher, R. J. H. Greig, A. Bueno-Orovio, B. Rodriguez, Human in silico drug trials demonstrate higher accuracy than animal models in predicting clinical pro-arrhythmic cardiotoxicity. *Front. Physiol.* **8**, 668 (2017).

53. A. Muszkiewicz, O. J. Britton, P. Gemmell, E. Passini, C. Sánchez, X. Zhou, A. Carusi, T Alexander Quinn, K. Burrage, A. Bueno-Orovio, B. Rodriguez, Variability in cardiac electrophysiology: Using experimentally-calibrated populations of models to move beyond the single virtual physiological human paradigm. *Prog. Biophys. Mol. Biol.* **120**, 115–127 (2016).

54. M. Zaniboni, I. Riva, F. Cacciani, M. Groppi, How different two almost identical action potentials can be: A model study on cardiac repolarization. *Math. Biosci.* **228**, 56–70 (2010).

55. M. P. Adams, E. J. Y. Koh, M. P. Vilas, C. J. Collier, V. M. Lambert, S. A. Sisson, M. Quiroz, E. McDonald-Madden, L. J. McKenzie, K. R. O'Brien, Predicting seagrass decline due to cumulative stressors. *Environ. Modelling Software* **130**, 104717 (2020).

56. T. Cui, X. Tong, O. Zahm, Prior normalization for certified likelihood-informed subspace detection of Bayesian inverse problems. arXiv:2202.00074 [math.NA] (31 January 2022).

57. D. Fernández Slezak, C. Suárez, G. A. Cecchi, G. Marshall, G. Stolovitzky, When the optimal is not the best: Parameter estimation in complex biological models. *PLOS ONE* **5**, e13283 (2010).

58. A. Doucet, S. Godsill, C. Andrieu, On sequential Monte Carlo sampling methods for Bayesian filtering. *Stat. Comput.* **10**, 197–208 (2000).

59. M. K. Transtrum, P. Qiu, Optimal experiment selection for parameter estimation in biological differential equation models. *BMC Bioinformatics* **13**, 181 (2012).

60. B. Schölkopf, A. Smola, K.-R. Müller, Nonlinear component analysis as a kernel eigenvalue problem. *Neural Comput.* **10**, 1299–1319 (1998).

61. T. Pavlenko, A. Björkström, A. Tillander, Covariance structure approximation via gLasso in high-dimensional supervised classification. *J. Appl. Stat.* **39**, 1643–1666 (2012).

62. C. Tönsing, J. Timmer, C. Kreutz, Cause and cure of sloppiness in ordinary differential equation models. *Phys. Rev. E* **90**, 023303 (2014).

63. S. Kleinegesse, C. Drovandi, M. U. Gutmann, Sequential Bayesian experimental design for implicit models via mutual information. *Bayesian Anal.* **3**, 773–802 (2021).

64. C. Beisbart, N. J. Saam, *Computer Simulation Validation: Fundamental Concepts, Methodological Frameworks, and Philosophical Perspectives* (Springer, 2019).

65. C. E. Dangerfield, D. Kay, K. Burrage, Modeling ion channel dynamics through reflected stochastic differential equations. *Phys. Rev. E* **85**, 051907 (2012).

66. J. Grazzini, M. Richiardi, Estimation of ergodic agent-based models by simulated minimum distance. *J. Econ. Dyn. Control.* **51**, 148–165 (2015).

67. S. A. Sisson, Y. Fan, M. A. Beaumont, *Handbook of Approximate Bayesian Computation* (Chapman & Hall/CRC Press, 2018).

68. L. F. Price, C. C. Drovandi, A. Lee, D. J. Nott, Bayesian synthetic likelihood. *J. Comput. Graph. Stat.* **27**, 1–11 (2018).

69. A. Beskos, A. Jasra, K. Law, Y. Marzouk, Y. Zhou, Multilevel sequential Monte Carlo with dimension-independent likelihood-informed proposals. *SIAM/ASA J. Uncertain. Quantif.* **6**, 762 (2018).

70. U. K. Müller, Measuring prior sensitivity and prior informativeness in large Bayesian models. *J. Monetary Econ.* **59**, 581–597 (2012).

71. E. J. Milner-Gulland, K. Shea, Embracing uncertainty in applied ecology. *J. Appl. Ecol.* **54**, 2063–2068 (2017).

72. W. K. Newey, D. McFadden, *Handbook of Econometrics* (Elsevier, 1994), vol. 4, pp. 2111–2245.

73. K. M. Banner, K. M. Irvine, T. J. Rodhouse, The use of Bayesian priors in ecology: The good, the bad and the not great. *Methods Ecol. Evol.* **11**, 882 (2020).

74. C. P. Robert, G. Casella, *Monte Carlo Statistical Methods* (Springer-Verlag, 1999).

75. M. I. Jordan, Z. Ghahramani, T. S. Jaakkola, L. K. Saul, An introduction to variational methods for graphical models. *Mach. Learn.* **37**, 183–233 (1999).

76. L. Tierney, J. B. Kadane, Accurate approximations for posterior moments and marginal densities. *J. Am. Stat. Assoc.* **81**, 82–86 (1986).

77. J. B. Tenenbaum, V. de Silva, J. C. Langford, A global geometric framework for nonlinear dimensionality reduction. *Science* **290**, 2319–2323 (2000).

78. T. Cui, K. J. H. Law, Y. M. Marzouk, Dimension-independent likelihood-informed MCMC. *J. Comput. Phys.* **304**, 109–137 (2016).

79. M. Evans, H. Moshonov, Checking for prior-data conflict. *Bayesian Anal.* **1**, 893 (2006).

80. S. G. Walker, Bayesian inference with misspecified models. *J. Stat. Planning Inference* **143**, 1621–1633 (2013).

81. J. D. Hoffman, S. Frankel, *Numerical Methods for Engineers and Scientists* (CRC Press, 2018).

82. H. M. Bücker, G. Corliss, P. Hovland, U. Naumann, B. Norris, *Automatic Differentiation: Applications, Theory, and Implementations* (Springer-Verlag, 2006), vol. 50.

83. P. G. Constantine, C. Kent, T. Bui-Thanh, Accelerating Markov chain Monte Carlo with active subspaces. *SIAM J. Sci. Comput.* **38**, A2779 (2016).

84. T. Cui, X. T. Tong, A unified performance analysis of likelihood-informed subspace methods. arXiv:2101.02417 [stat.CO] (7 January 2021).

85. O. Zahm, T. Cui, K. Law, A. Spantini, Y. Marzouk, Certified dimension reduction in nonlinear Bayesian inverse problems. arXiv:1807.03712 [math.PR] (2 July 2022).

86. A. W. Bowman, A. Azzalini, *Applied Smoothing Techniques for Data Analysis: The Kernel Approach with S-Plus Illustrations* (Oxford Univ. Press Inc., 1997), vol. 18.

87. M. C. Jones, Simple boundary correction for kernel density estimation. *Stat. Comput.* **3**, 135–146 (1993).

88. S. Dokos, *Modelling Organs, Tissues, Cells and Devices: Using MATLAB and COMSOL Multiphysics* (Springer, 2017).
